# Round the Hospitals

**Published:** 1921-08-13

**Authors:** 


### ROUND THE HOSPITALS.

In our issue of July 30 the appointment was announced of Miss Forster-Feather to the matronship of Addenbrooke's Hospital, Cambridge. Two days after accepting the appointment we are informed that Miss Forster-Feather asked to be allowed to withdraw, and a special meeting of the Hospital Committee was held on August 1 to elect another lady as matron in her stead. Miss A. C. Bell was then unanimously appointed matron in succession to Miss Crookenden. Miss Bell was trained at St. Thomas's Hospital, and on the completion of her training in 1914 was appointed ward-sister to the Royal Hospital for Sick Children, Edinburgh, succeeding later to the post of night sister and home sister at the same institution. Appointed assistant matron to the General Hospital, Nottingham, in April 1918, she held that post till April 1920, when she was selected as matron to the Herefordshire General Hospital, which post she at present holds. We offer Miss Bell our congratulations on her appointment to Addenbrooke's.

Miss Cox-Davies, we are glad to report, is continuing to make steady progress towards recovery. She has been much touched and cheered by the numerous expressions and tokens of sympathy which she has received from members of the nursing profession and others, for which she is very grateful.

A medical man recently said that if he were a nurse he would like to belong to the Royal Naval Nursing Service, and on being asked for his reason explained that as it was such a small Service its members would, without doubt, be particularly well treated. We were reminded of this on reading the new rates of pay, leave, and pensions for members of this Service, which have been issued. We do not think they can be said to err on the side of over-generosity. The new rates of pay, inclusive of bonus, and to take effect as from April 1 last, unless sisters should prefer to continue to draw their old rates of pay with bonus up to the present date, are:—Head sisters, £170, rising by £10 annually to £200; superintending sisters, £95, rising by £5 to £135; nursing sisters, £60, rising by £5 to £85. Head sisters have seven weeks' leave of absence annually, superintending sisters six weeks, and nursing sisters five weeks for the first three years and six weeks thereafter; in all cases full pay is paid during leave. The maximum rates of retired pay, which is made up of a service element and a rank element, are head sister £160, superintending sister £105, nursing sister £75; sisters retiring with less than ten completed years' service receive a gratuity equivalent to one month's pay in respect of each year of service.

The Marquis and Marchioness of Camden entertained the members of the Kent Nursing Association at Bayham Abbey on July 30 with singularly delightful hospitality. The setting for the large gathering, which included nurses from all parts of Kent and even eight candidates in training at Plaistow, was extremely beautiful, and all the arrangements were made with most thoughtful consideration for the nurses' pleasure and comfort. In this the proceedings were in marked contrast to similar gatherings we have witnessed, in which the guests have been bidden to tea on bare benches and trestle-tables in the blazing sun and invited to refresh themselves afterwards with a game of rounders. A touch of imagination on the part of the hostess, Lady Camden, who is President of the Kent Nursing Association, turned an ordinary annual event into a festivity worthy of grateful remembrance.

On new regulations dealing with probationers at the Brentford Infirmary being submitted to the Guardians recently, an unsuccessful attempt was made by one of the Labour members to get the clause altered which gives the Hospital Committee power to call upon a nurse to resign without assigning any reason. He feared attempts might be made to get rid of nurses who displayed too much interest in their trade union. The proposal to limit the powers of the Committee to suspension and give to the full Board the onus of decision would

**ROUND THE HOSPITALS**—(*continued*).

inevitably introduce great confusion. It can surely be understood that there may be young women whose presence in a ward proves to be actually mischievous, yet against whom no definite charge of misconduct could be sustained.

---

The nurses' wing of the Cripples' Hospital at Hartshill, which is the hospital of the North Staffs Cripples' Aid Society, was opened by Lady Stafford at a garden-party which brought together a large gathering of friends and sympathisers with the objects of the Society. There is ample evidence to show the admirable work carried on in the institution, and it is universally recognised that it cannot properly fulfil its aims without a contented, well-housed nursing staff. The block now put at their disposition was originally intended for an operating theatre, but the urgent need for giving better accommodation to the nurses prevailed, and the theatre is the next requirement. The bedrooms in the nurses' home have been charmingly furnished by various friends, among whom are Lord and Lady Stafford. The proceeds of the opening festivities will suffice to furnish the remainder of the rooms. We hope to learn the hospital has been affiliated as a preliminary training-school for probationers.

---

The new Nurses' Home at the Herefordshire General Hospital is approaching completion. An interesting feature is the flat roof, which is to be arranged for the nurses' rest and recreation. In the original plan it was to be bounded only by a low wall, but we learn this is to be supplemented by an iron railing to provide for safety. A few movable wind screens in two folds to give shelter in "variable airs," are indispensable in all roof gardens which are to be of practical use. The neglect of such provision renders the flat roof a very doubtful advantage.

---

Nine of the Bagthorpe nursing sisters, under the Nottingham Board of Guardians, have had their war bonus decreased by £7, but at the same time an addition of £10 a year has been made to their salary, on the recommendation of the Nursing Staff Committee. The increase was sharply challenged by some members of the Board, but was agreed to by thirty votes to three. The opposition gave occasion for a warm eulogy of the nursing staff on the part of several of the guardians, male and female, one of whom declared that nothing was too great to give to women who were doing this work for the emancipation of their fellow-creatures.

---

A correspondent has asked us a question which appears to be exercising the minds of a considerable number of persons. She desires to be "put right" as to the payment of unemployment insurance on behalf of hospital sisters. She is correct in her assumption that they are insurable under the Act, provided their salaries, with emoluments, do not exceed £250, and this ruling applies to all classes of sisters —*i.e.*, ward sisters, theatre sisters, out-patient sisters, massage sisters, etc. In short, the position of sisters is the same as it is under the National Health Insurance Acts, unemployment insurance contributions being payable in respect to any for whom health insurance contributions are required to be paid. The Ministry of Labour will not express any general opinion as to the value to be placed on the emoluments (*i.e.*, board and lodging, etc.) received in addition to salary. As under the Health Insurance Acts, each case must be considered on its merits, having regard to the circumstances and conditions of employment, and where any doubt is felt as to whether health insurance contributions are payable an inquiry on the subject should be addressed to the Insurance Department of the Ministry of Health, Buckingham Gate, London, S.W. 1.

---

The licences of three massage establishments were cancelled at a special meeting of the L.C.C. Public Control Committee, held on July 22. In one case the County Council inspector, a woman, reported upon the conduct of a nurse. The proprietress admitted that the nurse was culpable, but said that she had immediately discharged her, and that she was prepared to carry on the establishment without assistance. Her licence was, however, cancelled, the Chairman insisting that those who conducted such establishments must take complete responsibility. It is evident that this very necessary inspection and control is being carried out with energy and skill in the public interest. The appeal of Miss Lucy Hichens against an order of the L.C.C. revoking her licence to carry on a massage establishment in Piccadilly was heard before Sir Charles Biron at Bow Street Police Court, on July 25, and was dismissed, with eighteen guineas costs.

---

Twenty-four years ago there were three district nurses in Huddersfield; to-day there are eighteen —one more illustration to explain why nurses are "scarce." At the annual meeting of the Huddersfield and District Victoria Sick Poor Nurses' Association it was reported that it now costs £10 a day to keep the work going. The births attended numbered 482 without one death. Thirteen people lend their cars for the nurses on Sundays when the trams do not run, and Dr. Moore, M.O.H., suggests that a motor-car or two might well be provided for their sole use.

---

The maternity needs of York City appear to be growing apace and to be well met. Nearly a thousand more cases were dealt with at the dispensary in connection with the Maternity Hospital last year than in 1919. The average cost per patient has gone down by 1s. 3d. A new maternity hospital is in course of erection and is much needed, as may be seen by the readiness of patients to pay the fees asked and something over. It was a record year of applications, but the organisation is so good that not one abnormal case had to be refused.

